# Hope in Pediatric Palliative Care: A Systematic Review

**DOI:** 10.3390/children13081117

**Published:** 2026-08-21

**Authors:** Hana Benešová, Miroslava Janoušková, Martin Loučka

**Affiliations:** 1 Department of Psychiatry, First Faculty of Medicine, Charles University and General University Hospital, 128 08 Prague, Czech Republic; 2 Paediatric Supportive Care Team, Motol and Homolka University Hospital, 150 06 Prague, Czech Republic; 3 Department of Psychiatry and Medical Psychology, Third Faculty of Medicine, Charles University, 116 36 Prague, Czech Republic; 4 Department of Medical Psychology and Ethics, Faculty of Medicine, Masaryk University, 601 77 Brno, Czech Republic

**Keywords:** hope, palliative care, pediatrics, parents, child, infant, adolescent, siblings

## Abstract

**Highlights:**

**What are the main findings?**

• We have identified key factors that can foster or diminish hope in children with life-threatening illnesses and their families.
• We show that hope is not just an individual trait, but is associated with external factors—many of which are modifiable.

**What are the implications of the main findings?**

• Healthcare providers should be trained and encouraged to communicate honestly but empathetically, actively support family hope.
• Another implication is the need for comprehensive family-centered care: supporting siblings, providing respite to caregivers, addressing financial questions via social work, and integrating spiritual care.

**Abstract:**

**Context**: Previous studies and reviews in adult patients have shown that hope is an important coping mechanism in patients with serious health problems. Although there are individual studies in pediatric patients, there is a lack of a systematic review of how hope manifests and how it can be supported in children with life-threatening illnesses and their families. **Objectives**: This systematic review aims to identify the key factors that are associated with the experience of hope in pediatric palliative care. It examines how hope is fostered or diminished in children with life-limiting illnesses, their parents, and other family members. **Methods**: A systematic search was conducted across major medical databases for studies published between 1990 and 2024. Thematic analysis was used to identify factors associated with hope. The quality of the included studies was critically appraised. **Results**: Out of 1373 identified records, 105 studies met the inclusion criteria. Most studies were qualitative in design and met a high proportion of the applicable criteria in their respective design-specific appraisal tools. Factors associated with increased hope included open and empathetic communication, trusting relationships with healthcare providers, strong social and family support, spiritual and religious faith, effective symptom management, involvement in care decisions, and access to practical resources. Conversely, reduced hope was associated with poor communication, social isolation, uncontrolled symptoms, uncertainty, caregiver distress, financial strain, and negative healthcare experiences. These factors were consistent across cultural and diagnostic contexts, with some variation in emphasis. **Conclusions**: Hope in pediatric palliative care is associated with relational, psychological, spiritual, and systemic factors. Many of these are modifiable. A family-centered and multidisciplinary approach is essential to sustaining hope in pediatric palliative contexts.

## 1. Introduction

A serious illness in a child is a significant crisis situation that places many demands on the entire family system and has far-reaching consequences for the mental health of the child patients themselves, their parents, siblings, as well as the extended family [1,2]. Hope represents an important coping mechanism for all involved in these situations, even in the face of dire prognoses and terminal stages of illness [3,4,5,6]. Since most parents wish to be fully informed about the health status of their sick child, it seems reasonable to inform parents about the prognosis truthfully while leaving space for hope [7,8,9,10,11]. Parents and child patients draw hope from various sources [12], and when providing healthcare and psychosocial care, it is necessary to inquire about the family and identify individual sources of hope [13].

Targeted work with hope is exemplified in the recommended practices of the American Academy of Pediatrics for providing pediatric palliative care [9]. Healthcare staff may worry that activating the palliative care team will take hope away from families [14]. Recent work with adult patients shows that involving the palliative care team does not diminish hope; on the contrary, it can help ‘recalibrate’ it [14,15] The study by Sisk [16] demonstrates that for most parents, hope for recovery is an important source of overall hope, remaining important even in stages of illness with poor prognosis. In its overall conclusions, the study identifies other sources and forms of hope, including the importance of maintaining quality of life, availability of quality care, and accessibility of doctors and nurses, which corresponds with the results of other studies as well [5,12]. Still, clinicians may be concerned that some factors (e.g., open communication or prognostic disclosure) will reduce hope in patients and their families [17].

The aim of this review is to summarize and connect our current knowledge about the factors associated with the experience of hope. Our review question is: What factors are associated with the experience of hope in pediatric patients in palliative care, their parents or other relatives?

## 2. Materials and Methods

### 2.1. Review Design

This systematic review is reported following the Preferred Reporting Items for Systematic Review and Meta-analysis Protocol (http://www.prisma-statement.org/, accessed on 10 June 2025), in compliance with the PRISMA guidelines. The PRISMA checklist is in Supplementary Table S1. This systematic review was not prospectively registered. The heterogeneity of study designs and their results precluded the possibility of meta-analysis.

### 2.2. Inclusion and Exclusion Criteria

#### 2.2.1. Population

We included studies on pediatric patients (children and adolescents, 0–19 years) and their family members who received palliative care during their illness. Although our eligibility criteria focused on pediatric participants, studies that also enrolled young adults were retained if they included children or adolescents within the predefined age range. Because all such studies contained data from the target pediatric population, they were considered eligible and were not excluded solely on the basis of including older participants. Similarly, studies with mixed participant samples that included healthcare professionals were eligible, but only if patients and/or caregivers were also represented and the reported findings were relevant to the experiences of these groups. Studies involving healthcare professionals as the sole participant group were excluded. We did not have geographical restrictions on the study population.

#### 2.2.2. Concept

We included original works of all types—qualitative and quantitative studies, and case reports.

#### 2.2.3. Settings

We searched for studies addressing hope and identifying variables that are associated with hope and its experiencing by the study population. Studies in which palliative care was not explicitly identified as the primary context were eligible only when the study population included children with life-threatening or life-limiting conditions and the reported findings addressed palliative-care-related aspects directly relevant to the review question.

Table 1 presents the inclusion and exclusion criteria for assessing the relevance of studies in relation to the topic of our research.

### 2.3. Searches

The search for this systematic review was conducted separately in PubMed, and in Academic Search Ultimate, APA PsycArticles, APA PsycInfo, MEDLINE Complete, and SocINDEX via EBSCO databases. We searched in all languages, within the data range from January 1990 to December 2024. At the same time, we were conducting follow-up searches on citations found in included studies. The search formula using relevant keywords and MeSH terms for all included databases is in Supplementary Table S2.

All search results were combined in the Rayyan platform (www.rayyan.ai) and duplicates were eliminated automatically. In the next phase, two researchers independently evaluated abstracts, determining whether to include them for further analysis. This process was completely manual. Discrepancies were then resolved by the two reviewers through discussion, with the assistance of a third researcher.

In preparing this review study, we also decided to include Large Language Models (LLMs). We used LLM ChatGPT-4o to analyze the full texts of studies (with the aim to help to decide whether to include certain studies, according to the criteria listed in Table 1), and to extract studied variables associated with hope. All results of the automated analysis were subsequently evaluated against full text of studies and rigorously manually edited by the main author of this publication. Therefore, the LLM served only as a “copilot” accelerating the work and preventing possible errors of oversight, as recommended by guidelines for the use of LLM in literature reviews [18,19,20].

### 2.4. Quality Appraisal

The methodological quality of the included studies was appraised using the design-specific critical appraisal tools developed by the Joanna Briggs Institute (retrieved from: https://jbi-global-wiki.refined.site/space/MANUAL, accessed on 10 June 2025). The appraisal was conducted by one reviewer (HB) and was not independently verified by a second reviewer. Each item was rated as “yes”, “no”, “unclear”, or “not applicable”. Items rated as “unclear” were treated as criteria not met, whereas “not applicable” items were excluded from the denominator.

For descriptive purposes, the number of criteria met was expressed as a percentage of all applicable items within the relevant checklist. The appraisal did not determine study inclusion and was not used to formally weight individual studies in the synthesis. All eligible studies contributed to the thematic synthesis, while study design and identified methodological limitations were considered when interpreting the findings.

### 2.5. Data Analysis

For consistent data extraction, we were inspired by the process described by Braun and Clarke [21] using semantic coding. The main author closely reviewed the results and findings sections of each included study and generated semantic codes capturing explicitly reported factors that were associated with increased or decreased hope among patients and their family members. The codes were kept as close as possible to the terminology and intended meaning of the original studies.

Findings from qualitative, quantitative, and mixed-methods studies were coded within the same thematic framework.

Factors were recorded in a structured coding matrix. Data extraction and initial semantic coding were performed by the main author (HB). A second reviewer (MJ) subsequently reviewed the coding matrix, category definitions, and emerging thematic structure. Uncertainties and differences in interpretation were discussed within the research team until consensus was reached. The thematic structure was developed iteratively, with codes and categories repeatedly reviewed for internal coherence, conceptual distinctiveness, and consistency with the underlying study findings.

Study design was considered during interpretation. Qualitative findings were mostly used to describe the meaning and context of each factor, whereas quantitative findings helped to indicate the direction and statistical support of the association, without directly comparing their relative strength.

When a finding did not fit the emerging framework, it was retained and discussed, and the framework was revised as necessary by modifying category definitions, reassigning codes, or creating a new category.

Due to the heterogeneity of source studies and the data reported, statistical analysis of data was not possible.

## 3. Results

**Figure 1:**
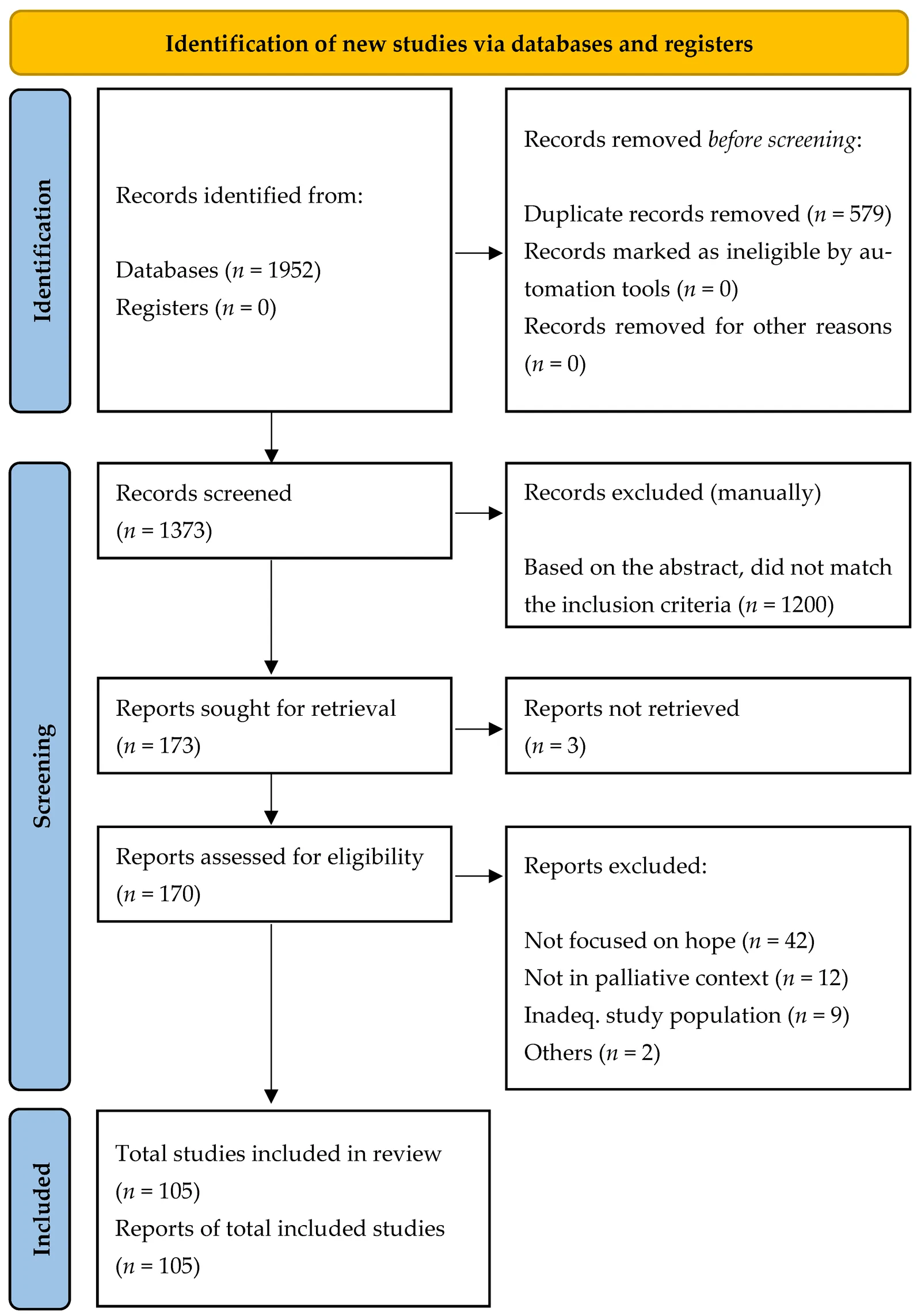
PRISMA 2020 flow diagram.

Out of 1373 articles identified by the search strategy, 173 underwent an abstract evaluation phase and after full-text evaluation, 105 articles [7,12,16,22,23,24,25,26,27,28,29,30,31,32,33,34,35,36,37,38,39,40,41,42,43,44,45,46,47,48,49,50,51,52,53,54,55,56,57,58,59,60,61,62,63,64,65,66,67,68,69,70,71,72,73,74,75,76,77,78,79,80,81,82,83,84,85,86,87,88,89,90,91,92,93,94,95,96,97,98,99,100,101,102,103,104,105,106,107,108,109,110,111,112,113,114,115,116,117,118,119,120,121,122,123] met the inclusion criteria (see Figure 1 for the PRISMA 2020 flow diagram).

A description of these studies is summarized in Table 2. Most were qualitative studies or interviews, and a minority were prospective or randomized trials. The number of participants varied from one (case studies) to 546; the median was 27 and the mean was 60.3. Most studies focused exclusively on family members or other caregivers (86/105, 81.9%), whereas only 9 studies (8.5%) included patients alone, and 10 studies (9.5%) enrolled both patients and family members. Siblings were rarely represented as study participants and only one study focused exclusively on bereaved siblings.

In the patient-only group, five studies were restricted to participants aged ≤19 years, while four also included young adults (upper age limits ranging from 24 to 30 years). Among studies enrolling both patients and family members, seven included only pediatric patients, while one extended recruitment up to 25 years of age.

Six studies included healthcare professionals alongside children and/or caregivers, whereas all remaining studies enrolled only pediatric participants, caregivers, or both.

In 15 articles (14.3%), it was not possible to consistently evaluate the JBI score; the remaining studies had a score above 70%, with the majority reaching above 85%.

### 3.1. Hope Measurement

In the case of quantitative studies, the main tools used for evaluation of hope were the Herth hope index (six studies) and the Trait hope scale (three studies). A detailed overview is given in Table 2.

**Table 2:** The characteristics of included studies (*N* = 105).

Author	Year of Publication	JBI Critical Appraisal Checklist Score	Study Design	Country	Number of Participants	Description of Participants	Method of Quantitative Evaluation of Hope (If Performed)	Codes Associated with Increased Experience of Hope	Codes Associated with Decreased Experience of Hope
Ahn et al. [22]	2018	100%	Qualitative, grounded theory, in-depth interviews	South Korea	12	Mothers of children with complex congenital heart disease		Successful curative treatment outcomes (in this case surgical); support from peer groups and healthcare providers; pride in child’s strength	Overwhelming caregiving burden; guilt; self-isolation; anxiety about child’s future; neglect of other children
Albuquerque et al. [23]	2024	100%	Qualitative, multiple case study, interviews	Brazil	18	Family members of 9 children eligible for pediatric palliative care		Faith in divine intervention; desire to return home; emotional support; proactive planning for care	Lack of communication; clinical deterioration; socioeconomic challenges; absence of clear health support structure
Atout et al. [24]	2019	100%	Qualitative, multiple case study	Jordan	48	Mothers of children (*n* = 15), physicians (*n* = 12), nurses (*n* = 21)		Protective communication; emotional support from caregivers	Open discussions about prognosis; lack of parental skills to communicate
Bally et al. [25]	2014	100%	Qualitative, grounded theory, interviews and journals	Canada	16	Parents (primarily mothers) of children in cancer treatment, aged 3–13 years		Seeing child healthy; being informed; feeling supported; gaining experience; spiritual or medical trust	Child’s suffering; lack of information; emotional isolation; uncertainty; spiritual doubts
Bally et al. [26]	2021	92%	Mixed methods, quasi-experimental crossover, interviews	Canada	58	Parents (mothers, fathers), grandparents, and one foster parent of children (3 months to 14 years) with LLI or LTI	Herth Hope Index (HHI)	Mindfulness; journaling; positive reframing; family activities; celebrating milestones	Overwhelm; isolation; lack of control; information overload
Barrera et al. [27]	2005		Qualitative, semi-structured interviews	Canada	12	7 mothers, 2 fathers, 3 children (7–15 years old) with incurable cancer		Belief in treatment efficacy; open communication; spiritual beliefs; focus on family time	Ambivalence about treatment; limited communication; awareness of physical decline
Barrera et al. [28]	2013	100%	Qualitative, interviews, grounded theory	Canada	35	Parents (26 mothers, 9 fathers) of children with poor cancer prognosis		Positive treatment response; spirituality; family/community support; focusing on present	Negative illness effects; information overload; negativity from others; exhaustion; fear; uncertainty
Bell et al. [29]	2019	91%	Quantitative, cross-sectional survey	United States	202	Biological mothers of children with Duchenne/Becker muscular dystrophy	Parent Hope Scale (PHS)	Spirituality; positive relationships; determination; resources; optimistic future orientation	Low spirituality; higher perceived uncertainty; lower confidence in coping
Bennett et al. [30]	2023	100%	Qualitative, grounded theory, interviews and document analysis	UK	13	Parents (*n* = 13) of children (aged 0–17) with life-limiting or life-threatening conditions; 3 bereaved		Timely and respectful conversations; written plans reflecting family values; shared understanding with clinicians; autonomy	Clinician avoidance; poor communication; exclusion from decisions; lack of plan implementation
Boateng et al. [31]	2024	90%	Qualitative (phenomenological, interviews)	Ghana	12	Family caregivers (mostly mothers, some grandmothers, aunt, sister, one father) of children with ESKD		Prayers; church attendance; support from relatives and health professionals; informative caregiving education	Financial burden; child’s deteriorating condition; lack of social support; stigma and spiritual interpretations (e.g., curses)
Boursange et al. [32]	2022	100%	Mixed methods (interviews + quantitative scales)	France	18	Parents of children with spinal muscular atrophy (SMA) type 1	Not directly evaluated; indirectly through interviews and psychological scales	Perceived physician confidence; positive treatment outcomes; access to innovative therapies; support from associations	Mixed treatment results; child’s medical complications; misinformation; pressure to decide quickly
Bronsema et al. [33]	2022	81%	Quantitative, cross-sectional survey	Germany	56	Bereaved parents of children who received specialist pediatric palliative care	FIN-PED (Family Inventory of Needs-Pediatrics) II—item: ‘to feel there was hope’	Opportunity to say goodbye; sincerity of professionals; being informed	Lack of time for self; unmet sibling support needs; lack of information
Callaghan [34]	2007		Qualitative, single-case study	Canada	1	14-year-old boy with life-limiting Ewing’s sarcoma		Participation in normal activities; control over symptoms; inclusion in care decisions; parental emotional support	Social isolation; treatment side effects affecting appearance; lack of peer contact
Carnevale [35]	2004		Qualitative case study (narrative/ethical commentary)	Canada		Family (parents) and a 16-year-old girl with terminal brain tumor		Prayer and spirituality; visible improvement in condition; support from trusted healthcare professionals; active role in treatment decisions	Direct and hopeless communication from medical staff; perception of staff resignation; lack of alignment in values and communication
Carr et al. [36]	2022	100%	Qualitative, interviews	United Kingdom and Republic of Ireland	17	Bereaved and non-bereaved parents of children with life-limiting/life-threatening conditions		Empathetic and respectful HCP approach; involvement in planning; clarity; feeling heard and valued; faith/spirituality	Poor timing of discussions; lack of trust; inadequate communication; absence of partner; HCP discomfort
Carroll et al. [37]	2012	90%	Qualitative, interviews	USA	73	Parents of 50 pediatric palliative care patients		Spirituality; sense of purpose; supportive healthcare relationships; hope for improvement; clear goals	Ambiguity in prognosis; perceived suffering of child; lack of understanding; emotional distress
Drouin et al. [38]	2024	75%	Quantitative, intervention (support group), pre-post evaluation	USA	36	Bereaved parents (76% women, avg. age 42.8) whose children died 6 months to 5 years earlier	Grief and Meaning Reconstruction Inventory (GMRI), Multicultural Quality of Life Index (MQLI), Feedback Survey	Peer connection; feeling heard; safe space to share; learning from others’ grief journeys	Limited session duration; lack of deeper discussion on key topics; absence of in-person connection
Dutta et al. [39]	2022		Randomized controlled trial (pilot)	Singapore	26	Parent-caregivers of children with chronic life-threatening illnesses		Affirmation from therapist; convenience of online writing; meaningful reflection; psychoeducation; flexible format of care	App technical issues, limited language accessibility of app; short duration of care; unclear questions
Effendy et al. [40]	2022	100%	Qualitative, semi-structured interviews	Indonesia	12	Parents (11 mothers, 1 father) of children with cancer who received home-based PPC		Education and training by nurses; emotional support; symptom management; logistic and financial aid	Cultural resistance to palliative care; feelings of guilt and failure; social stigma; initial fear of giving up curative treatment
Einarsdóttir [41]	2009		Qualitative, interviews	Iceland	53	28 mothers and 25 fathers of preterm infants with birth weight ≤1000 g		Faith in medicine; signs of infant improvement; support from medical staff; spiritual practices; naming rituals	Uncertainty of prognosis; lack of communication ability; anticipated severe disability
Erby et al. [42]	2006	100%	Qualitative, interviews	USA	17	Parents of sons with Duchenne Muscular Dystrophy, aged 7+ years		Belief in future treatments; meaningful milestones (e.g., college plans); presence in daily life	Avoidance of difficult topics; overwhelming caregiving demands; lack of professional guidance
Falkenburg et al. [43]	2020	90%	Qualitative, interviews	Netherlands	36	Parents whose children died in a PICU five years earlier		Perceived contact with the child; symbolic signs (butterflies, music, light); continuing bond	Social stigma or ridicule when expressing spiritual experiences; internal ambivalence; lack of religious framework
Feudtner et al. [44]	2010	80%	Quantitative, prospective cohort study	USA	43	Parents of pediatric patients receiving palliative care consultative services	Adult Dispositional Hope Scale	Higher positive affect; sense of agency and pathways to goals	Higher negative affect; perception of steep health decline
Fonseca et al. [45]	2021	90%	Qualitative, semi-structured interviews	Portugal	10	Parents (4 fathers, 6 mothers) of children with complex chronic conditions		Therapeutic letters; emotional support; affirmation of parental abilities; timely communication	Lack of emotional support; absence of recognition; poor communication timing
Gengler [46]	2015	90%	Qualitative, interviews and ethnographic observation	USA	18	Families of seriously ill children treated at a university research hospital		Restructuring social interactions; selective sharing of updates; avoiding emotionally draining conversations; support from emotionally affirming individuals	Discouraging questions from the surrounding community and medical staff; pessimistic reactions; emotionally taxing conversations; conflicting treatment suggestions
Ghoshal et al. [47]	2023	90%	Qualitative, interviews	India	20	Parents of children with advanced cancer		Honesty from clinicians (in this case oncologists); clear communication; inclusion of child in discussion; meaning-making	Vague communication; lack of empathy; uncertainty; excessive optimism; exclusion of child
Graetz et al. [48]	2021	100%	Qualitative, interviews, audio recordings	Guatemala	20	Parents of children newly diagnosed with cancer at UNOP		Psychologists’ use of metaphors and drawings; oncologists’ clear explanation of cure; support from staff and other families	Pre-existing fatalistic beliefs; prior community experiences with death; misinformation
Granek et al. [12]	2013	100%	Qualitative, longitudinal, grounded theory, interviews	Canada	35	Parents (26 mothers, 9 fathers) of children with cancer with poor prognosis		Positive treatment response; remission; quality time; spiritual beliefs	Disease progression; treatment failure; child’s visible suffering
Guon et al. [49]	2014	91%	Mixed methods (quantitative and qualitative survey)	USA, Canada, UK, and 12 other countries	128	Parents who continued pregnancy after prenatal diagnosis of trisomy 13 or 18		Respectful and personalized care; balanced information; acknowledgment of the child as a person	Pressure to terminate pregnancy; value-laden language; lack of support
Haase et al. [50]	2017	100%	Quantitative, confirmatory theory evaluation	United States	113	Adolescents and young adults (11–24 years) with cancer undergoing stem cell transplant	Hope-derived meaning scale (expectancy/interconnectedness and positive readiness)	Spiritual perspective; family communication; healthcare provider support; courageous coping	Illness-related distress (symptom distress, uncertainty); defensive coping
Harris et al. [51]	2023	100%	Qualitative, longitudinal, semi-structured interviews	USA	27	Pregnant mothers and support persons (mothers, fathers, others) after prenatal CHD diagnosis		Support from clinicians; repeated clear communication; spiritual belief; day-to-day focus; trust in providers	High mortality prognosis; lack of control; overwhelming information; poor hospital communication
Hendricks-Ferguson et al. [52]	2017	100%	Quantitative, pilot, single-group longitudinal	USA	13	Parents of children (aged 0.4–14.5) with brain tumor and poor prognosis	Herth Hope Index (HHI)	Tailored information sharing; nonabandonment messages; early prognosis discussion	Delayed or unclear communication about prognosis and PC/EOL
Hill et al. [53]	2018		Mixed-methods prospective cohort study	USA	199	Parents of seriously ill children (primary decision-makers)		Development of new domains over time; ongoing evaluation of child’s condition	Decline in domains like pain and suffering; reduced quality of life as death approaches
Hill et al. [54]	2017	91%	Mixed-method prospective cohort	USA	84	Parents of seriously ill children (42 parental dyads)		Improving condition; future-oriented goals; support for decision-making	Worsening illness; lack of consensus between parents; focus on immediate survival
Hill et al. [55]	2015	90%	Qualitative; semi-structured interviews	USA	110	71 parents (mothers, fathers, others) and 39 physicians of 50 children receiving palliative care		Quality of life; alleviation of suffering; improvements in physical condition; better medical knowledge; spiritual meaning	Poor communication; uncertainty in diagnosis; intractable symptoms; absence of shared understanding
Hinton et al. [56]	2017	100%	Qualitative, interviews	United Kingdom	31	Parents of children diagnosed with multiple sclerosis		Optimistic thinking; present-focused coping; belief in treatment improvements; avoiding negative information	Exposure to negative prognoses; conflicting medical opinions; visible relapses; unsupportive social interactions
Hjorth et al. [57]	2021	100%	Qualitative, narrative interviews and participant observations	Sweden	2 families	Children with SMA type 1 or 2 and their family members		Perceived physical improvement; possibility of future; support from family	Parental burnout; fear of disappointment; uncertainty about long-term treatment effects
Hullmann et al. [58]	2014	81%	Quantitative, survey	USA	85	Parents of children (ages 2–18) undergoing cancer treatment	Snyder’s Hope Scale	Cognitive flexibility; goal reappraisal; perceived social support; self-efficacy; meaning-making	Lower ability to find benefits in stressors; reduced goal-directed thinking; lack of social support
Chong et al. [59]	2021	100%	Qualitative multiple-case study	Singapore	22	8 bereaved parents, 14 health and social care professionals		Letting go; involving child in decisions; memory-making; supportive environment; symptom control; family togetherness	Loss of control; unmanaged symptoms; frequent hospitalizations; prolonged dying; disempowering environments
Chong et al. [60]	2023	100%	Qualitative, semi-structured interviews	Malaysia	13	Malaysian Muslim caregivers of children with ALL		Prayer; spiritual growth; belief in God’s plan	Uncertainty; emotional burden without spiritual support
Jalmsell et al. [7]	2016	87%	Qualitative, interviews	Sweden	10	Children aged 7–17 years with cancer in active treatment		Positive framing of bad news; inclusion in discussions; maintaining honesty; reassurance	False expectations; exclusion from information; unclear or overly simplified language
Janvier et al. [61]	2020	85%	Mixed-methods (questionnaire and thematic analysis)	USA, Canada, and 13 other countries	332	Parents of children with trisomy 13 or 18 who were part of online social support networks		Supportive communication; clinician presence; being given realistic hope; respect for family values	Use of dehumanizing language; lack of empathy; judgmental attitudes; pressure to make end-of-life decisions
Janvier et al. [62]	2016	91%	Quantitative, survey	USA, Canada, UK, other	261	Parents of children with full trisomy 13 or 18 (202 mothers, 59 fathers)	Self-reported questionnaire, open-ended and structured questions	Postnatal diagnosis; survival beyond expectations; child’s vitality	Prenatal diagnosis; medical pressure to terminate; perceived hopelessness
Kaemingk et al. [63]	2021	100%	Qualitative, interviews (audio-recorded consultations)	USA	25	Pregnant women anticipating delivery at 22–25 weeks’ gestation		Relationship building; acknowledgment and acceptance of uncertainty	Information overload; paralyzing uncertainty; lack of interaction during consultations
Kamihara et al. [64]	2015	90%	Qualitative, interviews	United States	32	Parents of children with relapsed or refractory cancer		Open clinician communication; acknowledgment of hopes beyond cure; daily meaningful moments; relational support	Lack of direct discussion about hope by clinicians; prognostic uncertainty; emotional dissonance; minimal space for parental expression
Kars et al. [65]	2010	88%	Qualitative (interviews)	Netherlands	44	Parents (23 mothers, 21 fathers) of 23 children with incurable cancer		Hope for cure or stabilization; professional support; spiritual beliefs; maintaining peaceful relationships; reframing by professionals	Certainty of incurability; clear signs of deterioration; perception of suffering; professional communication about end-of-life
Kiefer et al. [66]	2020	88%	Qualitative, longitudinal, semi-structured interviews	Germany	8	Caregivers (mainly mothers) of children with SMA Type 1 enrolled in nusinersen EAP		Good communication with medical staff; clinical improvement of child; transparency in treatment process	Lack of information on start/enrollment of specific modality of care; fear of exclusion from treatment; bureaucratic uncertainty
Kim et al. [67]	2019	100%	Quantitative, cross-sectional, structural equation modeling	South Korea	220	Adolescents aged 10–18 with a chronic health condition receiving treatment	Herth Hope Index	Social support	Illness-related distress
Kim et al. [68]	2021	86%	Quantitative, cross-sectional survey	South Korea	109	Parents of children (aged 7–18) undergoing cancer treatment	Good Death Inventory (GDI)—Korean version	Discussion of end-of-life care; agreement with child’s living will	Lack of discussion about end-of-life care; disagreement with establishing a living will
Koch et al. [69]	2023	88%	Qualitative descriptive study (interviews)	USA	46	Parents and healthcare providers (nurses, physicians, nurse practitioners) of 11 infants with complex congenital heart disease (CCHD)		Trusting relationships with providers; honest and frequent communication; emotional support	Poor communication; perceived hopelessness by providers; emotional isolation or judgment
Kolmar et al. [70]	2023	100%	Qualitative, semi-structured interviews	USA	10	Muslim parents of children with life-limiting conditions		Prayer; leaning into faith; support in religious practices; honest yet compassionate communication	Perceived discrimination; negative provider attitudes towards religion; overly clinical or blunt information delivery
Larson et al. [71]	1998	100%	Qualitative, in-depth case studies, interviews and participant observation	United States	6	Mexican-origin mothers of children with disabilities		Positive illusions; supportive maternal identity; optimistic expectations; perception of control	Medical pessimism; insensitive prognoses; systemic barriers; initial diagnosis
Lau et al. [72]	2019	90%	Randomized controlled trial (RCT), exploratory post hoc analysis	USA	99	Adolescents and young adults (AYAs) with cancer, aged 12–25		Resilience training; cognitive reframing; goal setting; benefit finding	Absence of structured psychosocial intervention (usual care only)
Layshock et al. [73]	2024		Qualitative, interviews	USA	19	Family caregivers of children with medical complexity (CMC)		Celebrating joy; support from family/friends/faith communities; moments for self-care	Lack of social support; emotional isolation; inability to process emotions; inability to make time for oneself
Lee et al. [74]	2020	85%	Qualitative, interviews	South Korea	12	Mothers of children with complex congenital heart disease (CHD) who had undergone heart surgery		Positive outcomes of curative treatment; improvements in child’s condition; support from others; information seeking	Feeling of abandonment; anxiety about losing the child; emotional burden of caregiving
Leite et al. [75]	2021	100%	Qualitative, narrative research, photo-elicitation interviews	Brazil	10	Family members of 3 children/adolescents with chronic illness (parents, siblings, aunts)		Support (emotional, spiritual, financial); faith; positive information; sense of normality; optimistic family members	Negative comparisons; fear of death; lack of clear information; invasive treatments; financial strain
Levine et al. [76]	2021		Quantitative, survey	USA	100	Pediatric oncology patients (age 10–18) and their parents, 1–12 months post-diagnosis	Original survey assessing perceived prognosis vs. objective prognosis	Positive communication; belief in high chance of cure; supportive clinician relationships	Realistic or pessimistic prognostic communication; diagnosis of leukemia
Levine et al. [77]	2020	100%	Qualitative, retrospective cohort, semantic content analysis	USA	100	Pediatric HCT patients and their families		Spirituality; resiliency; positive attitude; supportive family	Negative past experiences; lack of support; decisional doubt
Lin et al. [78]	2020	100%	Qualitative, descriptive phenomenological, interviews	Taiwan	3	Parents of 2 adolescents with brain stem dysfunction on life support		Spiritual practices; messages from deities; family rituals; perceived child responsiveness	Conflicting medical advice; lack of understanding; unfulfilled expectations; perceived suffering
Liu et al. [79]	2014	100%	Qualitative, descriptive, interviews	Taiwan	16	Parents of 11 children (7 mothers, 9 fathers); children aged 1 month to 18 years; PICU patients		Empathetic communication by healthcare staff; religious faith; parental belief in doing their best	Cold or routine communication by staff; guilt and regret; limited opportunity to say goodbye
Lotz et al. [80]	2017	88%	Qualitative interview study	Germany	11	Parents (8 mothers, 3 fathers) of 9 deceased children with life-limiting conditions		Open and honest communication; gradual planning; empowerment; planning for quality time; involvement in decisions	Physicians’ avoidance of discussions; prognostic uncertainty; overwhelming or premature planning; systemic disorganization
Lou et al. [81]	2015	95%	Qualitative (phenomenological, retrospective interviews)	Taiwan	10	Mothers of children who died from terminal brain tumor, members of CBTA-Taiwan		Spiritual beliefs; open discussions about death; support from family and healthcare professionals; meaningful rituals	Isolation; communication difficulties; emotional turmoil; helplessness; ambiguity about disease progression
Lövgren et al. [82]	2015	85%	Qualitative (content analysis of open-ended survey responses)	Sweden	108	Bereaved siblings of children who died from cancer, aged 19–33 at data collection		Being included in care; clear communication; playful information methods; support groups; empathy from HCPs	Exclusion from care; lack of information; perceived invisibility; routine/unempathetic HCP behavior
Mahoney et al. [83]	2023	100%	Qualitative, ethnographic fieldwork, semistructured interviews	Denmark	12	Parents of children (ages 10–18) with DMD or SMA type 2, plus one child and one assistant		Access to new treatments; participation in trials; physical therapy; sharing success stories	Lack of access to treatment; perceived futility; social isolation; ethical concerns about hope sharing
Maikranz et al. [84]	2007	100%	Quantitative, survey and electronic monitoring	USA	70	Pediatric renal and liver transplant recipients aged 7–18 and their caregivers	Children’s Hope Scale (CHS), Adult State and Trait Hope Scales	Better emotional adjustment; lower illness-related uncertainty	Higher levels of illness-related uncertainty; depressive symptoms
Manor-Binyamini et al. [85]	2022	100%	Qualitative, semi-structured interviews	Israel	15	Parents of children with terminal cancer, aged 2–18, in Israeli oncology ward		Family and social support; perception of hospital as a safe space; participation in research	Isolation; emotional exhaustion; conflicting feelings between acceptance and denial
Marron et al. [86]	2018	81%	Quantitative, survey	USA	353	Parents of children with newly diagnosed cancer	Parent survey on communication-related hope	High-quality communication style; high-quality prognostic information	Accurate understanding of poor prognosis
Martinez et al. [87]	2024	89%	Qualitative, semi-structured interviews	USA	27	Bereaved parents of children who died of cancer		Open communication with care team; flexibility in care; access to comfort care and chemotherapy	Lack of information; feeling of giving up; inadequate symptom management
Misko et al. [88]	2015	100%	Qualitative, interviews	Brazil	20 family members from 15 families	15 mothers, 3 fathers, 1 grandfather, 1 aunt of children/teenagers in palliative care		Spiritual beliefs; feeling supported by professionals; maintaining daily life and presence of child	Progression of symptoms; lack of support; physical and emotional exhaustion; child expressing desire to die
Moody et al. [89]	2020	90%	Pilot prospective single-arm study	USA	21	Parents of children with newly diagnosed cancer, some followed to end-of-life	Herth Hope Index	Early, empathic, honest goals-of-care discussions using complete intervention	Uncertainty; lack of empathic communication; absence of early goals-of-care planning
Nafratilova et al. [90]	2018	90%	Qualitative, descriptive phenomenology, interviews	Indonesia	10	Parents (8 mothers, 2 fathers) of children with cancer in palliative care		Belief in God; prayer; gratitude; spiritual support; emotional closeness	Poor communication with palliative care team; lack of coordination; ongoing despair
Nahalla et al. [91]	2003	100%	Qualitative, phenomenological, interviews	Sri Lanka	10	Parents of children with thalassaemia undergoing regular hospitalizations for blood transfusions		Kindness of medical staff; organized activities for children; access to regular transfusions; parental peer support	Financial difficulties; lack of medical resources; inadequate hospital facilities; social stigma
Nicholas et al. [92]	2017	100%	Qualitative, longitudinal grounded theory, interviews	Canada	35	Parents (26 mothers, 9 fathers) of children with poor-prognosis cancer		Spiritual connection; prayer; guidance; emotional decompression; support from religious groups	Spiritual depletion; perceived abandonment by God; emotional exhaustion; disrupted faith practices
Polita et al. [93]	2023	100%	Qualitative, narrative research, interviews	Brazil	13	Fathers of children (0–19 years) with recurrent cancer		Religious faith; spiritual meaning-making; peer support from others with similar experience; re-engagement with daily life	Disease recurrence; clinical deterioration; awareness of limited treatment efficacy; internalized emotional suppression
Popp et al. [94]	2015	81%	Quantitative, interviews and questionnaires	United States	50	Parents of children (under 18) receiving treatment for pediatric cancer	Trait Hope Scale	Resolution of diagnosis; higher family functioning; supportive perception of care	Unresolved status; emotional disengagement; preoccupation with diagnosis
Pourghaznein et al. [95]	2018	100%	Qualitative, hermeneutic phenomenology, interviews	Iran	11	Mothers of children undergoing hemodialysis		Successful treatment (in this case transplant) outcomes in other children; communication with healthcare professionals	Organ rejection; feeling ignored by medical staff and family; financial burden; witnessing child’s suffering
Prins et al. [96]	2023	90%	Qualitative, inductive analysis of audio-recorded conversations	Netherlands	68	Parents of 12 critically ill children in NICU/PICU and 46 physicians		Acknowledgment by physicians; affect-oriented responses; signs of clinical improvement	Cognition-oriented responses without emotional support; medical uncertainty
Rafferty [97]	2015		Qualitative (grounded theory, interviews)	United States	35	Parents of children receiving ongoing treatment for complex chronic conditions		Supportive communication; religious/spiritual beliefs; online and interpersonal information seeking; reframing narratives	Overwhelming obstacles; anticipatory grief; waiting endlessly; questioning the future
Rafferty et al. [98]	2020	100%	Qualitative, interviews, directed content analysis	United States	35	Parents (28 mothers, 7 fathers) of children with complex chronic conditions; mostly married, white, Christian, college-educated		Information from medical staff and peers; online support groups; spiritual beliefs; positive narratives	Uncertainty; lack of prognosis; unsupportive social environment; absence of information
Reggio et al. [99]	2021	100%	Qualitative, interviews	USA	37	Caregivers (mostly mothers) of children with incurable cancer or serious illness in ICU		Altruistic motivation; self-reflection; emotional support; structured study design	Sad memories evoked during interviews; emotional exhaustion
Robert et al. [100]	2022	86%	Qualitative (narrative analysis, interviews)	USA	7	Bereaved parents (4 mothers, 3 fathers) of children who died from cancer		Honest but hopeful communication; preparing for end of life; supporting child autonomy; team alignment	Lack of anticipatory guidance; conflicting messages from care team; avoidance of end-of-life conversations
Rosenberg et al. [101]	2018	86%	Prospective longitudinal mixed-methods study	USA	37 AYAs, 40 parents	Adolescents and Young Adults (14–25 years) with newly diagnosed non-CNS cancer and their parents	Dispositional Hope Scale (Agency and Pathway sub-scores)	Higher baseline hope; lower baseline distress; supportive psychosocial environment	Higher baseline psychological distress; progressive disease
Rujimora et al. [102]	2023	100%	Qualitative, interviews	United States	10	Adolescents and young adults (13–30 years) enrolled in a palliative peer-support program		Support from relatable peers; cultural/language matching; lasting relationships; activities and entertainment	Isolation; lack of peer interaction; emotional distress; absence of cultural/language alignment
Salins et al. [103]	2022	100%	Qualitative, interviews, thematic analysis	India	28	Adolescents aged 12–19 years with cancer attending a tertiary care center		Parental and sibling support; peer reassurance; spiritual faith; acceptance of illness; positive thinking	Fear; isolation; disfigurement; information concealment; societal misunderstanding
Samsel et al. [104]	2024	91%	Quantitative, cross-sectional survey	USA	27	Fathers of children hospitalized with advanced heart disease	Survey on prognosis understanding and communication	Open and honest communication; inclusion in clinical discussions; feeling heard	Conflicting information; limited prognostic clarity; underestimation of long-term limitations
Samson et al. [105]	2009	100%	Qualitative, phenomenological, interviews	Canada	12	Parents of children with Duchenne muscular dystrophy		Parental adaptation; active caregiving; child’s well-being; spiritual reflection	Initial diagnosis shock; passivity; perception of illness as external destructive force
Shi et al. [106]	2024	100%	Quantitative, cross-sectional survey, structural equation modeling	China	247	Caregivers of children with chronic kidney disease	Herth Hope Index (Chinese version)	Stable occupation; higher education; sufficient income; frequent online consultation; less traffic pressure	Delayed treatment; low income; low education; high stress; limited access to medical services
Schwartz-Attias et al. [107]	2024		Quantitative, cross-sectional survey	Israel	26	Bereaved parents of children who died of cancer (age 1–24)	Indirectly via Needs Assessment of Family Caregivers-Cancer (NAFC-C)	Presence with child; involvement in care; family and social support	Child’s suffering; inadequate psychosocial support; financial difficulties; unmet expectations from providers
Sisk et al. [108]	2020		Qualitative, interviews	USA	78	Parents of children with cancer (treatment, survivorship, or bereavement phase)		Demonstrating intent to cure; highlighting positives; redirecting to broader hopes	Overly blunt or false reassurances; lack of individualized communication
Sisk et al. [109]	2022	94%	Qualitative; semi-structured interviews	United States	37	Adolescents and young adults (12–24 years at diagnosis) with cancer, during treatment or survivorship		Positive clinician attitude; truthful but sensitive communication; support for individual goals (e.g., normalcy, friendships)	False reassurance; excessive optimism; lack of clarity; exclusion from communication
Sisk et al. [16]	2018	91%	Quantitative, longitudinal survey	United States	374	Parents of children recently diagnosed with cancer	Self-report questionnaires using Likert-scale questions	Hope for child’s well-being (cure, love, quality of life); perceived support from clinicians; parental agency; spiritual and social support	Information overload; emotional and physical exhaustion; uncertainty; lack of support from oncologist
Smith et al. [110]	2018	100%	Delphi study (qualitative and quantitative)	Canada, Australia, New Zealand	68	Parental caregivers, nurses, physicians, social workers, community members	Delphi method using Keeping Hope Possible framework	Connecting with others; Access to relevant information; Positive thinking; Celebrating milestones; Parental participation in care	Lack of support networks; Unmet basic needs; Unclear caregiver roles; Inadequate information
Soto Jansson et al. [111]	2024		Qualitative (interviews)	Sweden	36	Caregivers (biological and foster parents) of children with Dravet syndrome aged 1–19 years		Supportive hospital staff; support groups; access to respite care; shared experiences with other DS families	Uncertainty about future care; fear of premature death; lack of societal understanding; caregiver exhaustion
Spurr et al. [112]	2022	85%	Quantitative quasi-experimental study	Canada	58	Parents and caregivers of children with life-threatening or life-limiting illness	Herth Hope Index (HHI)	Staying at home with child; having no financial concerns; caregiving role prioritized	Working outside the home; financial concerns; absence of other children in household
Szabat et al. [113]	2020	100%	Qualitative, blog analysis, grounded theory, Colaizzi’s method	Poland	1	Parent (mother) of a child with Trisomy 18		Spiritual beliefs; family and social support; professional care; reframing normality; personal coping strategies like blogging	Inappropriate communication from healthcare providers; child’s deteriorating health; persistent uncertainty
Tewani et al. [114]	2023	100%	Qualitative, semi-structured interviews	Singapore	15	Mothers who chose to continue pregnancy despite life-limiting fetal condition		Religious belief; supportive family; coordinated perinatal care	Healthcare fragmentation; insensitive communication; lack of continuity
Tomlinson et al. [115]	2011		Cross-sectional, quantitative	Canada	26	Parents of children with cancer and no reasonable chance of cure	VAS (0–10) for importance of hope in decision making	Perceived potential for prolonged survival and improved QoL	Lack of consensus between parents; poor prognosis understanding
Truitt et al. [116]	2012	90%	Quantitative, survey	USA	546	Primary caregivers of children with Down syndrome	Trait Hope Scale (Snyder); Hope for child (modified version)	Goal-setting; support groups; planning ways to reach goals	Difficulty in planning ways to reach goals; high perceived uncertainty
van der Geest et al. [117]	2015	75%	Quantitative, survey	Netherlands	89	Parents of 57 children who died of cancer (mothers and fathers)	Custom questionnaire developed for the study	Meaningful time with child; child as coping source; support from healthcare professionals and family	Low spiritual reliance; minimal impact of faith or prayer
Vindrola-Padros et al. [118]	2017	90%	Qualitative, narrative interviews, participant observation	Argentina	17	Parents of children with cancer undergoing treatment in Buenos Aires		Securing accurate diagnosis and good treatment; proximity to family; returning home	Fear of relapse; distrust in local care; separation from home and daily life
von Scheven et al. [119]	2021		Qualitative, focus groups and workshops	USA	39	14 patients and 25 caregivers of children with chronic illnesses		Confidence in medical team; joyful clinical environment; empathetic providers; community connection; peer support	Poor communication; lack of empathy from providers; stigmatizing language; uncertainty
von Scheven et al. [120]	2024	91%	Mixed-methods, think-aloud cognitive interviews	USA	11	Youth aged 12–16 years with various chronic health conditions		Engaging in enjoyable activities; having dreams for the future; feeling supported; maintaining identity beyond illness	Physical limitations; pain; lack of energy; interference of illness with daily activities
Wakefield et al. [121]	2023		Mixed-methods (quantitative + interviews)	Australia	182 parents, 23 patients	Parents of children with high-risk cancer; adolescent patients aged 12–17	Validated questionnaire items and thematic analysis of interviews	Belief in helping future patients; expectation of more treatment options; sense of agency in decision-making	Long wait for results; disappointing or no treatment recommendations; lack of communication
Wong et al. [122]	2008	91%	Quantitative, survey	Australia	35	Parents of children aged 5–12 with cystic fibrosis	Vicarious Futurity Scale (VFS)	Emotional support; high vicarious hope	Self-blame; behavioral disengagement; high vicarious despair
Zelcer et al. [123]	2010	90%	Qualitative, focus group interviews	Canada	25	Parents of 17 children who had died of brain tumors		Social support; continuing daily routines	Loss of communication; lack of community support; symptom burden

### 3.2. Factors Associated with Hope

In the reviewed studies, we identified 346 individual codes associated with increased experience of hope and 305 individual codes associated with decreased experience of hope in children in palliative care and their families. Based on the codes, 28 unique factors associated with increased experience of hope and 30 unique factors associated with decreased experience of hope were formulated. Factors were grouped into 9 and 8 thematic groups, respectively, presented in the following paragraphs. All unique codes, factors and group of factors are also listed in Supplementary Table S3.

### 3.3. Factors Associated with Increased Hope

#### 3.3.1. Open Communication and Information Sharing

Clear and honest communication with healthcare providers has been shown to be a cornerstone of hope [47,69,70,80]. Parents and family members gain hope from early information, from prognostic discussions that balance realism with optimism, and from feeling that their questions are being answered openly [82,87].

#### 3.3.2. Trusting Relationships with Healthcare Providers

In addition to the content of communication, the quality of relationships with healthcare professionals is also crucial [51,69]. Families feel hopeful when they perceive the healthcare team as caring, present, and supportive [37,41,62]. A trusting and empathetic relationship reassures parents that they are not alone, thereby reinforcing hope [49,59,82].

#### 3.3.3. Family and Social Support & Togetherness

The majority of studies highlighted the importance of social support from family, friends and community as a crucial source of hope [27,85,94,107,113]. Strong family cohesion has been repeatedly identified as a factor that promotes hope. Parents gain hope from the presence of other family members who can share caregiving responsibilities and provide emotional comfort [31]. The possibility of staying at home with the child has also been highlighted in a number of studies as a factor that increases hope [49,59].

#### 3.3.4. Spiritual and Religious Faith

Spirituality and faith-based practices are powerful sources of hope for many families and appear in a variety of cultural contexts. Parents often cited religious faith (belief in God or a higher plan) as helping them cope and find hope even in difficult situations [60,70,90]. Praying, participating in religious ceremonies, or receiving support from faith communities were mentioned as specific ways families strengthened their hope [31,35,92].

#### 3.3.5. Effective Symptom Management and Comfort

Families experience greater hope when a child’s physical suffering is reduced and their comfort is maximized. Many studies have noted that effective symptom control (good pain management, relief from distressing symptoms) increases hope [35,55,87,96]. Similarly, any signs of clinical improvement in a child can generate hope [59,74,96].

#### 3.3.6. Involvement, Control and Empowerment

Active participation in the child’s care and decision-making process is another recurring theme that is associated with reinforced hope. Families feel more hopeful when they feel they can do something meaningful for their child [42,47,117]. Being treated as a valued member of the care team and having their voice heard in decisions (such as treatment choices and end-of-life plans) empowers parents and inspires hope [36,80,104].

#### 3.3.7. Positive Outlook and Coping Strategies

Many of the studies have highlighted that families that maintain an optimistic mindset or use active coping tend to have greater hope [7,71,75,77,103]. Examples of these factors include positive thinking, finding benefits, resilience, and determination. Cognitive reframing techniques (seeing situations in a more optimistic light) and belief in the child’s inner strength or vitality have been reported to be helpful [25,62]. Consciously building self-efficacy and self-confidence is also associated with increased hope [58].

#### 3.3.8. Meaning-Making and Legacy Activities

Finding meaning and purpose in the terrain of illness may be another way to strengthen hope. Many parents find hope in engaging in meaningful activities with their child. This can create positive memories, which then serve as useful factors in strengthening hope. This can include celebrating milestones (birthdays, holidays, even small achievements), taking family photos, or participating in meaningful rituals [25,110,117]. These activities give families a sense of legacy and continuity and strengthen the hope that their child’s life, however short, has meaning and will be remembered [37,117]. Opportunities to say goodbye properly or fulfill a child’s wishes were also cited as hope-enhancing factors [33].

#### 3.3.9. Financial and Practical Stability

Although financial and practical stability are less frequently discussed than psychosocial factors, several studies have identified these as important factors enabling hope [40,75,112]. When families have financial security or assistance, they can focus on caring for their child with less anxiety, thereby maintaining room for hope and for other hope fostering factors [40,107].

### 3.4. Factors Associated with Decreased Hope

#### 3.4.1. Information Gaps and Conflicting Messages

Communication breakdowns have been shown to be a major obstacle to maintaining hope. Families reported that lack of information, unclear explanations, or conflicting messages from healthcare providers left them frustrated, anxious, and less hopeful [46,56,78,100,104]. Conflicting information was particularly damaging because it created confusion and distrust. Unclear or technical language can also reduce hope. When families do not fully understand their child’s condition or what to expect, they may develop inadequate fears of the future [66,75,103].

#### 3.4.2. Social Isolation

Several studies have noted that social isolation has been linked to feelings of overwhelm and hopelessness in parents [22,27,85,88,120]. This includes situations where extended family or friends withdraw, leaving primary caregivers without a support network. Parents who have no time for themselves, are unable to work, and have little interaction outside of caregiving often report a diminishing sense of hope as their world shrinks [110,111]. For siblings, being excluded from family conversations or hospital visits has led to feelings of isolation that have diminished hope [82].

#### 3.4.3. Child’s Deteriorating Condition and Suffering

Factors such as “the child’s deteriorating condition,” “visible suffering,” “worsening symptoms,” and frequent health crises were commonly cited as factors that reduce hope [12,25,37,53,65,95]. This is why maintaining hope is often most challenging in the final stages of illness, unless balanced by acceptance or reframed goals (e.g., hoping for comfort rather than cure) [65,113].

#### 3.4.4. Uncertainty and Loss of Control

Not knowing what will happen or when is a psychologically draining condition. High uncertainty related to the disease has been linked to anxiety and a reduced sense of hope, as families cannot envision the future or set clear goals [25,50,55,63,64]. This goes hand in hand with a perceived loss of control, as the parents feel that nothing they do can change the outcome [26,51,59]. Bureaucratic or systemic uncertainty (such as long waits for test results or transplant availability) also contributes to this category [91,97,114,121].

#### 3.4.5. Emotional Distress and Mental Exhaustion

Emotions such as fear, anxiety, depression, guilt, and grief can be overwhelming. Many studies have noted that parents who experienced high levels of anxiety about their child’s future or fear of their child’s death often had difficulty maintaining hope [22,40,79,103]. This can become a vicious cycle: as hope diminishes, people often feel more despair, which further diminishes hope. Interventions to break this cycle (through counseling, support groups, respite measures, etc.) are key to preventing families from spiraling into hopelessness [22,82,111].

#### 3.4.6. Caregiver Overload

The practical burden of caring for a terminally ill child is definitely a challenging situation. In many countries, treatment for serious illnesses is often expensive [91,95,107,112], and several studies have found that financial problems are an additional source of stress, making it difficult for parents to focus on the hopeful aspects of life [31,112]. Along with financial problems, the logistical burden of caregiving can also be exhausting (frequent hospital visits, caring for other children, work, and constant caregiving). All of this can lead to caregiver burnout, which has often been cited as a factor that reduces hope [22,57,74].

#### 3.4.7. Negative Encounters with Healthcare System

Past and current negative experiences within the healthcare system or with healthcare providers can undermine hope. For example, a qualitative report on parents of infants with chromosomal trisomy described many families encountering dehumanizing language and judgmental attitudes from healthcare staff [113]. It is not only lack of empathy, but also system-related factors in healthcare, such as fragmentation or lack of continuity of care, bureaucracy, and delays of care support, that may be linked with diminished hope [23,91,113,114,121].

#### 3.4.8. Cultural and Spiritual Distress

Certain cultural or spiritual factors can negatively impact hope, particularly when families experience a crisis of faith or cultural conflicts in care. Perceived abandonment by God, spiritual doubts or a sense of punishment (feeling cursed or spiritually judged) can replace hope with despair [31,92]. Families also may face cultural barriers that diminish hope: stigma from their community, or cultural resistance to concepts like palliative care (e.g., when cultural backgrounds judge palliative care as “giving up”) [31,32,40,43,87].

## 4. Discussion

Our analysis provides a comprehensive overview of factors associated with the experience of hope in pediatric palliative care. We identified distinct factors associated with higher and lower levels of hope, highlighting the complexity of hope as a phenomenon that represents an important source of support for families in pediatric palliative care.

The preceding paragraphs have listed the dominant factors associated with the experience of hope. They show a high degree of convergence across studies. On closer inspection, some differences and nuances do emerge when considering subgroups and contexts.

Certain factors were more emphasized in specific cultural or regional contexts. For instance, religious faith and prayer were particularly salient in studies from countries with strong religious traditions (e.g., Ghana, Brazil, Malaysia, parts of the USA) [31,60,75,77,97,98]. Stigma and societal attitudes also varied by region: in some developing regions, families faced blame or gossip that hurt hope, whereas in most Western contexts, stigma was less about curses and more about isolation or lack of understanding from others [31,40,91,119].

Studies further differ in the populations studied. The majority of included studies are focused on parents, but a few show other perspectives, such as from bereaved siblings. The siblings’ perspective is aligned with many parental themes (they too wanted honest communication, involvement, and support), but siblings placed even greater emphasis on inclusion [75,82].

Further differences stem from the stage of the disease. In early or uncertain stages, many parents try to hope for a cure or long-term survival. As illness progresses and prognosis becomes poor, parents tend to redefine hope—hoping for quality of life, pain relief, or meaningful time rather than a cure [59,64,65,72].

Comparing pediatric and adult palliative populations, some notable differences can be found. Parents often feel a duty to “keep hope alive” for their child’s sake, even if personally they are exhausted [88]. In adult palliative patients, the individual’s own hope is paramount and may involve personal existential goals, for example to die at home. Adult palliative patients often emphasize peer support (e.g., support from friends or other patients) and not wanting to be a burden on family as factors in hope [124]. Regarding spirituality, our findings show that faith was a major comfort for parents, and spiritual crises could devalue hope [91]. Serious illness of their child can be an acute challenge to their previously held beliefs (“Why would God allow an innocent child to suffer?”). Adult patients, facing their own mortality, also grapple with existential questions, but some find solace in having lived a life or in afterlife beliefs [124,125,126].

Another comparison may be between parents (families) in palliative care programs and parents of children with non-palliative chronic illnesses. For parents of children with chronic but not immediately life-threatening illnesses (e.g., severe disabilities), hope factors often include advocating for the child, witnessing the child’s progress (even small milestones) [127], and receiving community inclusion [128]—partly overlapping with the themes identified in our review on palliative patients. In fact, hope might be one bridging concept across many caregiving situations: whether a child is terminally ill or has lifelong disabilities, parents need hope to cope, and they draw it from support, faith, information, and small victories.

The alignment between our findings and the other literature mentioned above underlines the importance of a few key practices. Healthcare providers should be trained and encouraged to communicate honestly but empathetically, actively supporting family hope (for example, by highlighting achievable goals or positives even while delivering tough news). Thus, the goal is a balance: a “hoping for the best, preparing for the worst” approach. Another implication is the need for comprehensive family-centered care: supporting siblings, providing respite to caregivers, addressing financial questions via social work, and integrating spiritual care. The multidisciplinary care team (including physicians of various specializations, nurses, social workers, chaplains, psychologists) is ideally positioned to target each domain of hope—physical, emotional, social, spiritual.

Despite a robust understanding of many hope factors, there remain areas for further inquiry. One recommended direction is to explore interventions that systematically enhance hope in pediatric palliative care. While the adult literature has tested hope-fostering interventions (such as dignity therapy, hope therapy, construct of “reasonable hope”, etc.) [129,130,131], in pediatrics, this is still yet emerging. Our findings suggest that multidisciplinary collaboration is the key. It seems reasonable to prospectively evaluate the contribution and effectiveness of such specifically built and trained multidisciplinary teams on fostering hope. Another research area is longitudinal studies of hope trajectories. As noted, hope is dynamic; understanding how families’ hopes evolve from diagnosis through end of life (and even into bereavement) could inform at which points certain supports are most needed. For instance, do families experience a “hope gap” when transitioning from curative to palliative intent? If so, targeted interventions at that transition (like a hope-focused consultation or a narrative intervention to help reframe hope) could be developed. Additionally, research could delve deeper into the child’s perspective on hope. We mostly extrapolate from parents, but direct voices would be valuable to tailor interventions.

It is important to acknowledge that our synthesis is only as robust as the studies underlying it. Many studies had small sample sizes or specific cultural settings, which may limit generalizability. Included studies have, perhaps even surprisingly, a relatively homogeneous direction in their conclusions. There is a possibility of publication bias: studies that identified clear “factors” might be more likely to be published, whereas those that found ambiguous results might be underrepresented. Our thematic categorization, although derived from the data, also inevitably involves subjective judgment. Most involved studies show parental reports of hope, with relatively fewer direct reports from children, and due to the small number of studies with pediatric patients as respondents, a separate sub-analysis was not performed. Most studies were from high-income countries in North America, Europe, and parts of Asia, with fewer from Africa or South America. Additionally, the concept of “hope” itself can be interpreted differently by participants. Some may think of hope in a more spiritual sense, others in a practical sense. In family therapy, the construct of “reasonable hope” exists, which may be a viable and reliable resource for clinicians with their work with clients [131]. The included studies did not typically aim to verify whether the hopes were reasonable/realistic, so we could not have explored this criterion of hope in our review. Due to different categorizations of patients into age groups across the methodologies of the included studies, our analysis did not deeply differentiate based on the child’s age. Studies that also enrolled young adults were retained if they included children or adolescents within the predefined age range. Similarly, studies including healthcare professionals alongside children and/or caregivers were retained, because all such studies contained data from the target population, although this may be a potential source of bias. Also, we included studies from both specialized and general palliative care settings based on the authors’ consensus during review process. However, we acknowledge that especially in general palliative care, where the definition is very wide, some additional, non-included studies could be seen relevant by other authors, and vice versa.

Despite these limitations, the convergence of findings across many studies lends credibility to our conclusions.

## 5. Conclusions

Hope is a multifaceted and vital component of the pediatric palliative care experience. Through our analysis of more than a hundred studies worldwide, we identified key factors that can nurture hope for children with life-limiting illnesses and their families, as well as factors that can diminish hope. Our findings show that hope is not merely an individual trait but strongly associated with external factors—many of which are modifiable. Supporting hope in pediatric palliative care requires a holistic approach. Medical teams must not only attend to the child’s physical symptoms but also foster open, compassionate communication and provide psychosocial and spiritual supports for the family. Interventions that strengthen family coping skills, connect families with peer support, and alleviate practical burdens are likely to pay dividends in maintaining hope.

## Figures and Tables

**Table 1:** Inclusion and exclusion criteria.

**Inclusion Criteria:**
peer-reviewed studies published between January 1990 and December 2024
studies addressing hope and variables that are associated with hope in pediatric patients in palliative care, in their parents, or other relatives
study types: all original works (qualitative and quantitative)
works written in any language
**Exclusion Criteria:**
studies that do not study hope directly from the perspective of patients or their relatives/caregivers, but examine it solely from the perspective of healthcare professionals or other persons involved in health/social care, etc.
studies, such as systematic reviews, editorials, theoretical articles, letters to the editor, and other article formats that do not process original data

## Data Availability

The study protocol is available from the corresponding authors on reasonable request due to laws and regulations.

## Supplementary Materials

The following supporting information can be downloaded at https://www.mdpi.com/article/10.3390/children13081117/s1, Table S1: Prisma 2020 Checklist; Table S2: MeSH terms and EBSCO formula; Table S3: Codes, factors and group of factors associated with experience of hope. Reference [132] are cited in the supplementary materials.

## Abbreviations

The following abbreviations are used in this manuscript:

ALL	Acute Lymphoblastic Leukemia
AYAs	Adolescents and Young Adults
CCHD	Complex Congenital Heart Disease
CHD	Congenital Heart Disease
ESKD	End-Stage Kidney Disease
HHI	Herth Hope Index
ICU	Intensive Care Unit
JBI	Joanna Briggs Institute
LLM	Large Language Model
MQLI	Multicultural Quality of Life Index
NICU	Neonatal Intensive Care Unit
PICU	Pediatric Intensive Care Unit
PHS	Parent Hope Scale
PRISMA	Preferred Reporting Items for Systematic Reviews and Meta-Analysis
QoL	Quality of Life
VAS	Visual Analog Scale
