# Supplementary material for: Hope in Pediatric Palliative Care: A Systematic Review
Source: Children (Basel). 2026 Aug 21;13(8):1117. doi: 10.3390/children13081117 (PMC13510848; doi:10.3390/children13081117)
Supplement: Supplementary file 1 [file children-13-01117-s001.zip › Table S2.pdf]

Table S2: MeSH terms and EBSCO formula

PubMed MESH terms:

("HopeOR hope OR hopefulness) AND (("Palliative Care"[MeSH] OR "Hospice and Palliative Care Nursing"[MeSH] OR palliative care OR hospice care OR end-of-life care OR terminal care OR serious illness OR life-threatening illness OR life-limiting condition* OR life-threatening condition* OR severe illness* OR chronic illness* OR terminal illness* OR neuromuscular disease* OR metabolic disorder* OR congenital anomaly* OR genetic disorder* OR progressive disease*) AND ("Parents"[MeSH] OR "Family"[MeSH] OR "Caregivers"[MeSH] OR "Next of Kin"[MeSH] OR parents OR caregivers OR family OR relatives OR siblings OR next of kin OR mother OR father)) AND ("Child"[MeSH] OR "Infant"[MeSH] OR "Adolescent"[MeSH] OR child OR children OR pediatric OR infant OR adolescent OR teenager) NOT ("Attitude of Health Personnel"[MeSH]) NOT ("Review"[Publication Type] OR "Editorial"[Publication Type] OR "Letter"[Publication Type])

EBSCO searching formula:

("Hope (Emotion)" OR hope OR hopefulness)
AND
(("Palliative Care" OR "Hospice Care" OR "Terminal Care" OR palliative care OR hospice care OR end-of-life care OR terminal care OR life-limiting condition OR life-threatening condition OR serious illness OR chronic illness OR progressive disease)
AND
("Parents" OR "Family" OR "Caregivers" OR "Siblings" OR parents OR caregivers OR family OR relatives OR siblings OR next of kin OR mother OR father))
AND
("Children" OR "Adolescents" OR "Infants" OR child OR children OR pediatric OR infant OR adolescent OR teenager)
NOT
("Health Personnel" OR "Nurses" OR "Physicians")
NOT
("Systematic Review" OR "Editorial" OR "Letter")
