# Supplementary material for: Hope in Pediatric Palliative Care: A Systematic Review
Source: Children (Basel). 2026 Aug 21;13(8):1117. doi: 10.3390/children13081117 (PMC13510848; doi:10.3390/children13081117)
Supplement: Supplementary file 1 [file children-13-01117-s001.zip › Table S3.pdf]

**Table S3: Codes, factors and group of factors associated with experience of hope**

| **Codes Associated with Increased Experience of Hope** |
|---|
| Acceptance of illness |
| Access to comfort care and chemotherapy |
| Access to innovative therapies |
| Access to medical care |
| Access to new treatments |
| Access to regular transfusions |
| Access to relevant information |
| Access to respite care |
| Acknowledgment and acceptance of uncertainty |
| Acknowledgment by physicians |
| Acknowledgment of hopes beyond cure |
| Acknowledgment of the child as a person |
| Active caregiving |
| Active role in treatment decisions |

| **Codes Associated with Decreased Experience of Hope** |
|---|
| Absence of clear health support structure |
| Absence of cultural/language alignment |
| Absence of early goals-of-care planning |
| Absence of information |
| Absence of in-person connection |
| Absence of other children in household |
| Absence of partner |
| Absence of recognition |
| Absence of shared understanding |
| Absence of structured psychosocial intervention (usual care only) |
| Accurate understanding of poor prognosis |
| Ambiguity about disease progression |
| Ambiguity in prognosis |
| Ambivalence about treatment |

| |
|---|
| Activities and entertainment |
| Affect-oriented responses |
| Affirmation from therapist |
| Affirmation of parental abilities |
| Agreement with child's living will |
| Alleviation of suffering |
| Altruistic motivation |
| Autonomy |
| Avoiding emotionally draining conversations |
| Avoiding negative information |
| Balanced information |
| Being given realistic hope |
| Being included in care |
| Being informed |
| Belief in future treatments |
| Belief in God |
| Belief in god's plan |

| |
|---|
| Anticipated severe disability |
| Anticipatory grief |
| Anxiety about child’s future |
| Anxiety about losing the child |
| App technical issues, limited language accessibility of app |
| Avoidance of difficult topics |
| Avoidance of end-of-life conversations |
| Awareness of limited treatment efficacy |
| Awareness of mortality |
| Awareness of physical decline |
| Behavioral disengagement |
| Bureaucratic uncertainty |
| Caregiver exhaustion |
| Certainty of incurability |
| Clear signs of deterioration |
| Clinical deterioration |
| Clinician avoidance |

| |
|---|
| Belief in helping future patients |
| Belief in high chance of cure |
| Belief in treatment efficacy |
| Belief in treatment improvements |
| Benefit finding |
| Better emotional adjustment |
| Better medical knowledge |
| Caregiving role prioritized |
| Celebrating joy |
| Celebrating milestones |
| Celebrating milestones |
| Child as coping source |
| Child’s vitality |
| Child’s well-being |
| Church attendance |
| Clarity |
| Clear communication |

| |
|---|
| Cognition-oriented responses without emotional support |
| Cold or routine communication by staff |
| Communication difficulties |
| Conflicting feelings between acceptance and denial |
| Conflicting information |
| Conflicting medical advice |
| Conflicting medical opinions |
| Conflicting messages from care team |
| Conflicting treatment suggestions |
| Cultural resistance to palliative care |
| Decisional doubt |
| Child expressing desire to die |
| Child’s deteriorating condition |
| Child’s deteriorating health |
| Child’s medical complications |
| Child’s suffering |
| Child's visible suffering |

| |
|---|
| Clear goals |
| Clinical improvement of child |
| Clinician presence |
| Cognitive flexibility |
| Cognitive reframing |
| Communication with healthcare professionals |
| Community connection |
| Confidence in medical team |
| Connecting with others |
| Continuing bond |
| Continuing daily routines |
| Control over symptoms |
| Convenience of online writing |
| Coordinated perinatal care |
| Courageous coping |
| Cultural/language matching |

| |
|---|
| Decline in domains like pain and suffering |
| Defensive coping |
| Delayed or unclear communication about prognosis and PC/EOL |
| Delayed treatment |
| Depressive symptoms |
| Diagnosis of leukemia |
| Difficulty in planning ways to reach goals |
| Direct and hopeless communication from medical staff |
| Disagreement with establishing a living will |
| Disappointing or no treatment recommendations |
| Discouraging questions from the surrounding community and medical staff |
| Disease progression |
| Disease recurrence |
| Disempowering environments |
| Disfigurement |
| Disrupted faith practices |

| | |
|---|---|
| Daily meaningful moments | Distrust in local care |
| Day-to-day focus | Emotional and physical exhaustion |
| Demonstrating intent to cure | Emotional burden of caregiving |
| Desire to return home | Emotional burden without spiritual support |
| Determination | Emotional disengagement |
| Development of new domains over time | Emotional dissonance |
| Discussion of end-of-life care | Emotional distress |
| Early prognosis discussion | Emotional exhaustion |
| Early, empathic, honest goals-of-care discussions using complete intervention | Emotional isolation |
| Education and training by nurses | Emotional isolation or judgment |
| Emotional closeness | Emotional turmoil |
| Emotional decompression | Emotionally taxing conversations |
| Emotional support | Ethical concerns about hope sharing |
| Emotional support from caregivers | Excessive optimism |
| Empathetic and respectful HCP approach | Exclusion from care |
| Empathetic communication by healthcare staff | Exclusion from communication |

| |
|---|
| Empathetic providers |
| Empathy from hcps |
| Empowerment |
| Engaging in enjoyable activities |
| Expectation of more treatment options |
| Faith |
| Faith in divine intervention |
| Faith in medicine |
| Faith/spirituality |
| Family activities |
| Family and social support |
| Family and social support |
| Family communication |
| Family rituals |
| Family togetherness |
| Family/community support |
| Feeling heard |

| |
|---|
| Exclusion from decisions |
| Exclusion from information |
| Exclusion of child |
| Exhaustion |
| Exposure to negative prognoses |
| False expectations |
| False reassurance |
| Fear |
| Fear of death |
| Fear of disappointment |
| Fear of exclusion from treatment |
| Fear of future complications |
| Fear of premature death |
| Fear of relapse |
| Feeling ignored by medical staff and family |
| Feeling of abandonment |
| Feeling of giving up |

| |
|---|
| Feeling heard and valued |
| Feeling supported |
| Feeling supported by professionals |
| Flexibility in care |
| Flexible format of care |
| Focus on family time |
| Focusing on present |
| Frequent online consultation |
| Future-oriented goals |
| Gaining experience |
| Goal reappraisal |
| Goal setting |
| |
| Good communication with medical staff |
| Gradual planning |
| Gratitude |
| Guidance |

| |
|---|
| Feelings of guilt and failure |
| Financial burden |
| Financial concerns |
| Financial difficulties |
| Financial strain |
| Focus on immediate survival |
| Frequent hospitalisations |
| Guilt |
| Guilt and regret |
| HCP discomfort |
| Healthcare fragmentation |
| Helplessness |
| High mortality prognosis |
| High perceived uncertainty |
| High stress |
| High vicarious despair |
| Higher baseline psychological distress |

| |
|---|
| Having dreams for the future |
| Having no financial concerns |
| Healthcare provider support |
| High vicarious hope |
| Higher baseline hope |
| Higher education |
| Higher family functioning |
| Higher positive affect |
| Highlighting positives |
| High-quality communication style |
| High-quality prognostic information |
| Honest and frequent communication |
| Honest but hopeful communication |
| Honest yet compassionate communication |
| Honesty from clinicians (in this cae oncologists) |
| Hope for cure or stabilization |
| Hope for child’s well-being (cure, love, quality of life) |

| |
|---|
| Higher levels of illness-related uncertainty |
| Higher negative affect |
| Higher perceived uncertainty |
| Illness-related distress |
| Inability to make time for oneself |
| Inability to process emotions |
| Inadequate communication |
| Inadequate hospital facilities |
| Inadequate information |
| Inadequate psychosocial support |
| Inadequate symptom management |
| Inappropriate communication from healthcare providers |
| Information concealment |
| Information overload |
| Initial diagnosis |
| Initial diagnosis shock |
| Initial fear of giving up curative treatment |

| |
|---|
| Hope for improvement |
| Improvements in child's condition |
| Improvements in physical condition |
| Improving condition |
| Inclusion in care decisions |
| Inclusion in clinical discussions |
| Inclusion in discussions |
| Inclusion of child in discussion |
| Information from medical staff and peers |
| Information seeking |
| Informative caregiving education |
| Involvement in care |
| Involvement in decisions |
| Involvement in planning |
| Involving child in decisions |
| Journaling |
| Joyful clinical environment |

| |
|---|
| Insensitive communication |
| Insensitive prognoses |
| Interference of illness with daily activities |
| Internal ambivalence |
| Internalized emotional suppression |
| Intractable symptoms |
| Invasive treatments |
| Isolation |
| Judgmental attitudes |
| Lack of access to treatment |
| Lack of alignment in values and communication |
| Lack of anticipatory guidance |
| Lack of clear information |
| Lack of communication |
| Lack of community support |
| Lack of consensus between parents |
| Lack of continuity |

| |
|---|
| Kindness of medical staff |
| Lasting relationships |
| Leaning into faith |
| Learning from others' grief journeys |
| Less traffic pressure |
| Letting go |
| Logistic and financial aid |
| Lower baseline distress |
| Lower illness-related uncertainty |
| Maintaining daily life and presence of child |
| Maintaining future plans |
| Maintaining honesty |
| Maintaining identity beyond illness |
| Maintaining peaceful relationships |
| Meaningful milestones (e.g., college plans) |
| Meaningful reflection |

| |
|---|
| Lack of control |
| Lack of coordination |
| Lack of deeper discussion on key topics |
| Lack of direct discussion about hope by clinicians |
| Lack of discussion about end-of-life care |
| Lack of emotional support |
| Lack of empathic communication |
| Lack of empathy from providers |
| Lack of energy |
| Lack of individualized communication |
| Lack of information |
| Lack of information on start/enrollment of specific modality of care |
| Lack of interaction during consultations |
| Lack of medical resources |
| Lack of parental skills to communicate |
| Lack of peer contact |

| |
|---|
| Meaningful rituals |
| Meaningful time with child |
| Meaning-making |
| Memory-making |
| Messages from deities |
| Mindfulness |
| Moments for self-care |
| Naming rituals |
| Nonabandonment messages |
| Normalization |
| Oncologists' clear explanation of cure |
| Ongoing evaluation of child's condition |
| Online and interpersonal information seeking |
| Online support groups |
| Open and honest communication |
| Open clinician communication |
| Open communication |

| |
|---|
| Lack of peer interaction |
| Lack of plan implementation |
| Lack of professional guidance |
| Lack of prognosis |
| Lack of religious framework |
| Lack of social support |
| Lack of societal understanding |
| Lack of support |
| Lack of support from oncologist |
| Lack of support networks |
| Lack of time for self |
| Lack of trust |
| Lack of understanding |
| Limited access to medical services |
| Limited communication |
| Limited opportunity to say goodbye |
| Limited prognostic clarity |

| | |
|---|---|
| Open communication with care team | Limited session duration |
| Open discussions about death | Long wait for results |
| Opportunity to say goodbye | Loss of communication |
| Optimistic expectations | Loss of control |
| Optimistic family members | Low education |
| Optimistic future orientation | Low income |
| Optimistic thinking | Low spiritual reliance |
| Organized activities for children | Low spirituality |
| Parental adaptation | Lower ability to find benefits in stressors |
| Parental agency | Lower confidence in coping |
| Parental and sibling support | Medical pessimism |
| Parental belief in doing their best | Medical pressure to terminate |
| Parental emotional support | Medical uncertainty |
| Parental participation in care | Minimal impact of faith or prayer |
| Parental peer support | Minimal space for parental expression |
| Participation in normal activities | Misinformation |
| Participation in research | Mixed treatment results |

| |
|---|
| Participation in trials |
| Peer connection |
| Peer reassurance |
| Peer support |
| Peer support from others with similar experience |
| Perceived contact with the child |
| Perceived child responsiveness |
| Perceived physical improvement |
| Perceived physician confidence |
| Perceived potential for prolonged survival and improved QoL |
| Perceived social support |
| Perceived support from clinicians |
| Perception of control |
| Perception of hospital as a safe space |
| Personal coping strategies like blogging |
| Physical therapy |
| Planning for quality time |

| |
|---|
| Negative comparisons |
| Negative illness effects |
| Negative past experiences |
| Negative provider attitudes towards religion |
| Negativity from others |
| Neglect of other children |
| Ongoing despair |
| Open discussions about prognosis |
| Organ rejection |
| Overly blunt or false reassurances |
| Overly clinical or blunt information delivery |
| Overwhelm |
| Overwhelming caregiving burden |
| Overwhelming caregiving demands |
| Overwhelming information |
| Overwhelming obstacles |
| Overwhelming or premature planning |

| |
|---|
| Planning ways to reach goals |
| Playful information methods |
| Positive attitude |
| Positive clinician attitude |
| Positive communication |
| Positive framing of bad news |
| Positive illusions |
| Positive information |
| Positive narratives |
| Positive outcomes of curative treatment |
| Positive parental attitude |
| Positive reframing |
| Positive relationships |
| Positive thinking |
| Positive treatment outcomes |
| Positive treatment response |
| Possibility of future |

| |
|---|
| Pain |
| Paralyzing uncertainty |
| Parental burnout |
| Passivity |
| Perceived abandonment by god |
| Perceived discrimination |
| Perceived futility |
| Perceived hopelessness |
| Perceived hopelessness by providers |
| Perceived invisibility |
| Perceived suffering |
| Perceived suffering of child |
| Perception of illness as external destructive force |
| Perception of staff resignation |
| Perception of steep health decline |
| Perception of suffering |
| Persistent uncertainty |

| |
|---|
| Postnatal diagnosis |
| Prayer |
| Prayers |
| Preparing for end of life |
| Presence in daily life |
| Presence with child |
| Present-focused coping |
| Pride in child’s strength |
| Proactive planning for care |
| Professional care |
| Professional support |
| Protective communication |
| Proximity to family |
| Psychoeducation |
| Psychologists’ use of metaphors and drawings |
| Quality of life |
| Quality time |

| |
|---|
| Pessimistic reactions |
| Physical and emotional exhaustion |
| Physical differences |
| Physical limitations |
| Physicians’ avoidance of discussions |
| Poor communication |
| Poor communication timing |
| Poor communication with palliative team |
| Poor hospital communication |
| Poor prognosis understanding |
| Poor timing of discussions |
| Pre-existing fatalistic beliefs |
| Prenatal diagnosis |
| Preoccupation with diagnosis |
| Pressure to decide quickly |
| Pressure to make end-of-life decisions |
| Pressure to terminate pregnancy |

| |
|---|
| Reassurance |
| Redirecting to broader hopes |
| Re-engagement with daily life |
| Reframing by professionals |
| Reframing narratives |
| Reframing normality |
| Relational support |
| Relationship building |
| Religious belief |
| Religious faith |
| Religious/spiritual beliefs |
| Remission |
| Repeated clear communication |
| Resilience training |
| Resiliency |
| Resolution of diagnosis |
| Resources |

| |
|---|
| Prior community experiences with death |
| Professional communication about end-of-life |
| Prognostic uncertainty |
| Progression of symptoms |
| Progressive disease |
| Prolonged dying |
| Questioning the future |
| Realistic or pessimistic prognostic communication |
| Reduced goal-directed thinking |
| Reduced quality of life as death approaches |
| Routine/unempathetic hcp behavior |
| Sad memories evoked during interviews |
| Self-blame |
| Self-isolation |
| Separation from home and daily life |
| Short duration of care |
| Social isolation |

| |
|---|
| Respect for family values |
| Respectful and personalized care |
| Restructuring social interactions |
| Returning home |
| Safe space to share |
| Securing accurate diagnosis and good treatment |
| Seeing child healthy |
| Selective sharing of updates |
| Self-efficacy |
| Self-reflection |
| Sense of agency and pathways to goals |
| Sense of agency in decision-making |
| Sense of normality |
| Sense of purpose |
| Shared experiences with other ds families |
| Shared understanding with clinicians |
| Sharing success stories |

| |
|---|
| Social stigma |
| Social stigma or ridicule when expressing spiritual experiences |
| Societal misunderstanding |
| Socioeconomic challenges |
| Spiritual depletion |
| Spiritual doubts |
| Stigma and spiritual interpretations (e.g. Curses) |
| Stigmatizing language |
| Symptom burden |
| Systemic barriers |
| Systemic disorganization |
| Teasing by peers |
| Treatment failure |
| Treatment side effects affecting appearance |
| Uncertainty |
| Uncertainty about future care |
| Uncertainty about long-term treatment effects |

| |
|---|
| Signs of clinical improvement |
| Signs of infant improvement |
| Sincerity of professionals |
| Social support |
| Spiritual and social support |
| Spiritual belief |
| Spiritual connection |
| Spiritual growth |
| Spiritual meaning |
| Spiritual meaning-making |
| Spiritual or medical trust |
| Spiritual perspective |
| Spiritual practices |
| Spiritual reflection |
| Spiritual support |
| Spirituality |
| Stable occupation |

| |
|---|
| Uncertainty in diagnosis |
| Uncertainty of prognosis |
| Unclear caregiver roles |
| Unclear or overly simplified language |
| Unclear questions |
| Underestimation of long-term limitations |
| Unfulfilled expectations |
| Unmanaged symptoms |
| Unmet basic needs |
| Unmet expectations from providers |
| Unmet sibling support needs |
| Unresolved status |
| Unsupportive social environment |
| Unsupportive social interactions |
| Use of dehumanizing language |
| Vague communication |
| Value-laden language |

| |
|---|
| Staying at home with child |
| Structured study design |
| Successful curative treatment outcomes (in this case surgical) |
| Successful treatment (in this case transplant) outcomes in other children |
| Sufficient income |
| Support (emotional, spiritual, financial) |
| Support for decision-making |
| Support for individual goals (e.g., normalcy, friendships) |
| Support from associations |
| Support from clinicians |
| Support from emotionally affirming individuals |
| Support from family |
| Support from friends/faith communities |
| Support from medical staff |
| Support from others |
| Support from peer groups and healthcare providers |

| |
|---|
| Visible relapses |
| Waiting endlessly |
| Witnessing child’s suffering |
| Working outside the home |
| Worsening illness |

| |
|---|
| Support from relatable peers |
| Support from religious groups |
| Support groups |
| Support in religious practices |
| Supporting child autonomy |
| Supportive clinician relationships |
| Supportive communication |
| Supportive environment |
| Supportive family |
| Supportive healthcare relationships |
| Supportive hospital staff |
| Supportive maternal identity |
| Supportive perception of care |
| Supportive psychosocial environment |
| Supportive relationships |
| Survival beyond expectations |
| Symbolic signs (butterflies, music, light) |

| |
|---|
| Symptom control |
| Symptom management |
| Tailored information sharing |
| Team alignment |
| Therapeutic letters |
| Timely and respectful conversations |
| Timely communication |
| Transparency in treatment process |
| Trust in providers |
| Trusting relationships with providers |
| Truthful but sensitive communication |
| Visible improvement in condition |
| Written plans reflecting family values |

| Factors and Groups of Factors Associated with Increased Experience of Hope | |
|---|---|
| **Groups** | **Factors** |
| **Open Communication and Information Sharing** | Clear and honest communication |
| | Early information |
| | Prognostic discussions |
| | Questions are being answered openly |
| **Trusting Relationships with Healthcare Providers** | Caring, present, and supportive team |
| | Not being alone |
| | Listen, “take their time,” and show compassion |
| | |

| Factors and Groups of Factors Associated with Increased Experience of Hope | |
|---|---|
| **Groups** | **Factors** |
| **Information Gaps and Conflicting Messages** | Lack of information |
| | Unclear explanations, conflicting messages |
| | Technical language |
| **Social Isolation** | Social Isolation |
| | Lack of support network |
| | Being excluded from family conversations |
| | Being excluded from hospital visits |

| | |
|---|---|
| **Family and Social Support & Togetherness** | "Companionship" with loved ones |
| | Possibility of staying at home with the child |
| | Presence of other family members |

| | |
|---|---|
| **Child's Deteriorating Condition and Suffering** | Visible suffering |
| | Worsening symptoms |
| | Health crises |
| | New complication |
| | Illness relapse |
| | Frequent hospitalizations |

| | |
|---|---|
| **Spiritual and Religious Faith** | Religious faith |
| | Praying |
| | Participating in religious ceremonies |
| | Receiving support from faith communities |

| | |
|---|---|
| **Uncertainty and Loss of Control** | Uncertainty related to the disease |
| | Perceived loss of control |
| | Bureaucratic or systemic uncertainty |

| | |
|---|---|
| **Effective Symptom Management and Comfort** | Effective symptom control |
| | Signs of clinical improvement |

| | |
|---|---|
| **Emotional Distress and Mental Exhaustion** | Overwhelming emotions such as fear, anxiety, depression, guilt, and grief |
| | Stress |

| | |
|---|---|
| **Involvement, Control and Empowerment** | Being treated as a valued member of the care team |
| | Active participation in the child’s care and decision-making process |

| | |
|---|---|
| **Caregiver overload** | Financial problems |
| | Logistical burden of caring |

| | |
|---|---|
| **Positive Outlook and Coping Strategies** | Optimistic mindset |
| | Active coping |
| | Building self-efficacy and self-confidence |

| | |
|---|---|
| **Negative Encounters with Healthcare System** | Dehumanizing language |
| | Judgmental attitudes from healthcare staff |
| | Fragmentation or lack of continuity of care |
| | Bureaucracy and delays of care support |

| | |
|---|---|
| **Meaning-Making and Legacy Activities** | Finding meaning and purpose |
| | Creating positive memories |
| | Celebrating milestones |
| | Sense of legacy and continuity |
| | Fulfill a child's wishes |
| | Opportunities to say goodbye properly |

| | |
|---|---|
| **Cultural and Spiritual Distress** | Cultural conflicts in care |
| | Spiritual doubts |
| | Sense of punishment |
| | Crisis of faith |
| | Stigma from their community |
| | Cultural resistance to concepts like palliative care |

| | |
|---|---|
| **Financial and Practical Stability** | Financial security or assistance |
